# Sex therapy for female sexual dysfunction

**DOI:** 10.1186/1755-7682-6-37

**Published:** 2013-09-26

**Authors:** Valeska Martinho Pereira, Oscar Arias-Carrión, Sergio Machado, Antonio Egidio Nardi, Adriana Cardoso Silva

**Affiliations:** 1 Panic & Respiration Laboratory, Institute of Psychiatry, Federal University of Rio de Janeiro, Rio de Janeiro, RJ, Brazil; 2 Movement Disorders and Transcranial Magnetic Stimulation Unit, Hospital General Dr. Manuel Gea Gonzalez, Secretaria de Salud, México, DF, México; 3 Institute of Phylosophy (IFILO), Federal University of Uberlândia (UFU), Uberlândia, Minas Gerais, Brazil; 4 Physical Activity Neuroscience, Physical Activity Postgraduate Program, Salgado de Oliveira University (UNIVERSO), Niterói, RJ, Brazil; 5 Quiropraxia Program of Faculty of Health Sciences, Central University (UCEN), Santiago, Chile; 6 National Institute for Translational Medicine (INCT-TM), Rio de Janeiro, Brazil

**Keywords:** Sexual dysfunctions, Psychological, Psychotherapy, Female, Behavior therapy, Cognitive therapy

## Abstract

**Introduction:**

About 45% of women suffer from some form of sexual dysfunction. Despite its high prevalence, there are few studies that have systematically evaluated sex therapy in comparison with other interventions.

**Objective:**

Review randomized clinical trials that present psychotherapeutic interventions for female sexual dysfunctions.

**Method:**

Through a search in three databases (Medline, Web of Science and PsycInfo), 1419 references were found. After an analysis of the abstracts, twenty-seven articles met the inclusion criteria and composed this review.

**Results:**

Sex therapy, as proposed by Masters and Johnson and Heiman and LoPiccolo, is still the most commonly used form of therapy for sexual dysfunctions; although it has shown results, the results do not consistently support that this is the best alternative in the treatment of sexual dysfunctions.

**Conclusion:**

There is a lack of systematic study of many female sexual dysfunctions. Orgasmic disorder and sexual pain (vaginismus and dyspaurenia) are the most extensively studied disorders and those in which sex therapy seems to have better outcomes.

## Introduction

Sexual dysfunctions (SD) are a common complaint among women, and it is estimated that 40 to 45% of adult women suffer from some form of sexual dysfunction [1]. The most frequently reported problems are desire and orgasmic dysfunction. A systematic review of prevalence rates have found a mean rate of 64% for desire problems; 35% for orgasmic difficulties; arousal problems, 31%; and for pain, 26% [2].

Masters and Johnson proposed a linear model to explain how humans respond to sexual stimuli. Their model is composed of four phases: excitement, plateau, orgasm and resolution [3]. Years later, desire was considered an important element in the human sex cycle [4]. The current model that serves as the basis for the classification and definition of sexual dysfunction is a combination of those two models and also has three phases: desire, arousal, orgasm.

Since sexual dysfunction treatment was proposed, the majority of clinical trials focused on orgasmic disorder, both primary (when women had never experienced orgasm by any means of stimulation) or secondary (women could achieve orgasm through self-stimulation but not in coitus). Anxiety was found to have an important role in sexual dysfunctions. The anticipation and performance anxiety could negatively impact sexual function. Based on that assumption, the main goal of sex therapy was to reduce the levels of anxiety related to sexual situations. Another goal was to improve sexual skills and repertoire [5,6].

Communication skills, listening exercises, emotional expression and reflection and conflict resolution are also important parts of treatment. This paper aims to review randomized clinical trials comparing psychological interventions to other forms of treatment in female population with sexual dysfunctions.

## Method

A literature search was conducted in three databases: Medline, Web of Science and PsycInfo, using the following keywords: female, sexual, dysfunction, clinical and trial. The search was conducted by two independent researchers in August 2013. A total of 1419 references were found (Pubmed, 1056; Web of Science, 200; Psycinfo, 163).

One hundred and eighty-one duplicated references were excluded, and 54 references in languages other than English or Portuguese were also excluded, leaving 1184 references to be evaluated by abstract analysis.

The inclusion criteria were as follows: 1. Randomized Clinical Trials comparing forms of treatment; 2. At least one psychotherapeutic intervention was used; and 3. Focus on female sexual dysfunctions (even when both men and women were treated).

**Figure 1:**
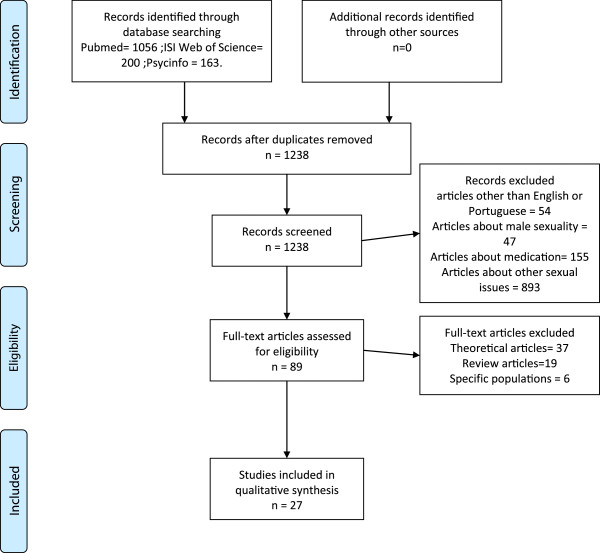
Flow diagram of selected studies.

After abstract analysis, 89 (eighty-nine) references were selected to full text analysis. Some articles did not meet the inclusion criteria and were excluded. This review was composed of 27 (twenty-seven) articles (for review, see Figure 1 and Table 1).

**Table 1:** Studies related to the effects of sex therapy on sexual dysfunction

**Study (Author/Year)**	**N**	**Objective**	**Conclusion**	**Reference**
Asirdas et al. 1975	23 (11 male and 12 female)	Systematic desensitization vs. classical conditioning.	14 subjects reported satisfaction after treatment, with better results in conditioning group.	[7]
Letourneau et al. 1997	25 women with Sexual Arousal Disorder	To create conditioned stimuli that could evoke sexual physiological and psychological responses.	The CS was not capable of evoking the same responses as the unconditioned stimulus.	[8]
Riley et al. 1978	20 women	Directed masturbation vs. sensate focus.	Masturbatory group were more capable of reaching orgasm.	[9]
Reisinger 1978	6 women	Masturbatory training associated with erotic stimulation.	The program was effective to reach orgasm.	[10]
Andersen 1981	30 primary nonorgasmic women	Directed masturbation vs. systematic desensitization	Masturbatory group were more capable of reaching orgasm.	[11]
Everaerd 1982	48 couples	Systematic desensitization vs. MJ (sensate focus and genital stimulation) vs. combined treatment.	MJ group had a faster response.	[12]
Crowe et al. 1981	48 couples	One with a couple of therapists vs. one with only one therapist.	Both groups were more interested in sex after treatment.	[13]
Everaerd et al. 1981	48 couples	MJ treatment vs. therapy focused on communication with no sexual interventions.	Women seemed to benefit from both forms of therapy, but sex therapy had more rapid results than communication therapy.	[14]
Kilmann et al. 1987	11 couples	Communication Skills plus Sexual skills vs. Sexual Skills plus Communication Skills.	No significant differences were found between the orders in which communication skills were presented.	[15]
Fichten et al. 1983	23 couples	Bibliotherapy with minimal therapist contact vs. classic couples therapy vs. group therapy.	Sensate focus and ban of intercourse led to an increase in enjoyment of non-coital sexual activities.	[16]
Libman et al. 1984	23 couples	Standard Couples Therapy (one hour per week with a therapist for 14 weeks) vs. Group Therapy (eight women, and partners only participated at three time points: beginning, middle and end of therapy) vs. bibliotherapy with minimal therapist contact.	All formats improved sexuality, but standard couples therapy had better results.	[17]
Fichten et al. 1986	23 couples	Standard Couples Therapy (one hour per week with a therapist for 14 weeks) vs. Group Therapy (eight women, and partners only participated at three time points: beginning, middle and end of therapy) vs. bibliotherapy with minimal therapist contact.	Affective and cognitive measures such as satisfaction and happiness had improved more than behavioral measures.	[18]
Morokoff et al. 1986	43 couples	Heiman and LoPiccolo manual in 2 groups: minimal therapist contact vs. full therapist contact.	The group with minimal therapist contact had better results.	[19]
Dodge et al. 1982	13 women	Heiman and LoPiccolo manual in 2 groups: minimal therapist contact vs. control.	Subjects improved in all measures and the gains were maintained in 6 month follow-up.	[20]
Spence 1985	50 women	Divided by primary or secondary orgasmic dysfunction in three conditions: group, individual e waiting-list control.	Both forms of treatment showed efficacy, but women with primary OD had worse outcomes.	[21]
Trudel et al. 1983	12 women	Kegel’s pelvic exercises vs. sexual awareness, respiratory training and muscle relaxation.	Kegel’s group didn’t show much improvement in sexual response.	[22]
Chambless et al. 1984	73 women	Kegel’s pelvic exercises vs. sexual awareness, respiratory training and muscle relaxation.	Kegel’s group didn’t show much improvement in sexual response.	[23]
Trudel et al. 2001	74 couples with hypoactive sexual desire disorder	CBT vs. waiting list control.	74% had remission of their symptoms.	[24]
Ter Kuile et al. 2007	117 women with vaginismus	CBT program vs. bibliotherapy vs. waiting list control group.	From 83 women in CBT group, 27 (33%) reported full penetration.	[25]
Masheb et al. 2009	50 women with vulvodynia	Compared CBT and supportive therapy.	Both approaches were effective, with 41.7% of women reporting a 33% reduction in pain scores.	[26]
Desrochers et al. 2010	97 women with vestibulodynia	CBT vs. topical treatment with cream.	Both groups showed improvements in pain scores, but at follow-up, the CBT group continued to show improvement.	[27]
van Lankeveld et al. 2001	199 couples	CBT bibliotherapy with minimum therapist contact by telephone vs. waiting list control group.	Treatment group had greater improvements and women with vaginismus seemed to have the greatest benefits from the program.	[28]
Mathews et al. 1983	48 couples	Combination of testosterone or placebo treatment; sex therapy with monthly or weekly sessions; a female therapist or female-male couple of therapists.	Better outcomes were in placebo plus weekly sessions.	[29]
Zimmer 1987	NA	Marital therapy and sex therapy vs. placebo and sex therapy	Both groups improved but marital group had more consistent results.	[30]
Jones et al. 2011	39 women	Evaluate an internet-based based on CBT.	Treatment group had improvements in sex life but not remission of symptoms.	[31]
Silverstein et al. 2011	44 (14 male and 30 female)	Evaluate mindfulness training.	Women showed faster responses to sexual stimuli.	[32]

## Results

The first clinical trial to evaluate behavioral treatment for sexual dysfunction compared systematic desensitization versus positive conditioning [7]. Both men (n = 11) and women (n = 12) were evaluated in physical, attitudinal and behavioral measures pre- and post-treatment. Systematic desensitization followed the model used in anxiety disorders. A hierarchy of scenes with sexual content was presented to the subjects along with techniques such muscle relaxation. Thus, the scenes were the same for all subjects, but the presentation order varied according to the patient classification. Positive conditioning consisted of the use of an unconditioned sexual stimulus paired with a neutral stimulus in order for it to become a conditioned stimulus capable of evoking the same responses as the unconditioned stimulus. In this study, women used a vibrator to achieve sexual excitement and they received a recorder with a male voice romantically describing a coitus scene. Before the vibrator use, women were instructed to fantasize about their sexual partner. In terms of results, both groups seemed to improve in all measures evaluated. Fourteen patients related good or very good attitudes toward sex after treatment. Two physical measures were evaluated: number of attempts to engage in sexual activity and the times these attempts were considered satisfying. For both groups, coital attempts and satisfaction increased. The authors noted that the conditioning group had better results.

Classical conditioning was used in another study, for female sexual arousal disorder [8]. The objective was to create conditioned stimuli that could evoke sexual physiological and psychological responses. The subjects were presented with erotic heterosexual movies that contained explicit intercourse and oral sex scenes. After the presentation of those movies excerpts, they were paired with a neutral stimulus, which was amber light. Classical conditioning theory proposes that smaller intervals between the two stimuli will lead to a stronger magnitude of conditioning. To evaluate this theory, two different conditions were created: E1 – with 10 seconds of interval; and E2 – a 1 second interval. Each subject received 50 sessions of conditioning, always one week after the end of menstrual period. To determine if the conditioning was successful, vaginal amplitude and subjective feelings of arousal were evaluated. After the 50 sessions, the conditioned stimulus (CS) was presented by itself to evaluate if it was capable of evoking the same response as the erotic videos. None of the experimental groups showed differences when compared to the control group. The CS was not capable of evoking the same responses as the unconditioned stimulus, so the study failed to demonstrate that female sexual arousal could be classically conditioned.

Some studies evaluated masturbatory training. One compared directed masturbation to conventional sex therapy, with sensate focus [9]. Both groups improved, but those that received masturbatory training obtained better results and were more capable of attaining orgasm through any means and during intercourse without the vibrator use. In another study [10], masturbatory training associated with erotic stimulation was found to be effective in orgasmic dysfunction. A third study compared directed masturbation to systematic desensitization [11], with results suggesting that women who received directed masturbation training not only had become orgasmic but also exhibited more variability in situations where they could reach orgasm.

Another study compared systematic desensitization, Masters and Johnson treatment (sensate focus and genital stimulation) and a combined treatment using both models [12]. The aim was to demonstrate that a combined treatment would be more effective, but the results did not corroborate this hypothesis. The findings reported that the Masters and Johnson treatment has a faster response compared to systematic desensitization. In a follow-up, the couples who had reduction in anxiety levels showed more consistent and permanent improvement.

The most studied treatment protocol was that of Masters and Johnson (MJ) [5]. Several clinical trials attempted to demonstrate the efficacy of it. In this treatment, sexual intercourse was banned for a period, sensate focus exercises were used, women on top position was recommended and the therapy was conducted by a couple of female and male therapists. One study attempted to investigate if those premises were fundamental and had an impact on outcomes [13]. Forty-eight couples presenting with sexual dysfunctions were assigned to two different groups: one with a couple of therapists and one with only one therapist. A third group was used as a comparison and had only one therapist, but the focus was on discussing marital problems. Both groups that received MJ therapy had become more interested in sex after treatment, but no statistical differences were found between the MJ groups and the control group. The number of therapists or their gender showed no influence on the results outcome.

Another study compared MJ treatment to a therapy focused on communication with no sexual interventions [14]. Their goal was to determine if improvement in couples’ communication could enhance sexual functioning. Communication therapy focused on active and passive listening, verbalization of and reflection on feelings, conflict management and assertive behavior. Forty-eight couples were randomly assigned to each group and 42 concluded treatment. The results showed that women seemed to benefit from both forms of therapy, but sex therapy had more rapid results than communication therapy. Sex therapy also produced better outcomes in women’s self-esteem.

Another study investigated whether the order in which communication skills were presented in therapy influenced treatment outcomes [15]. One group had Communication Skills plus Sexual skills; the other was the opposite. No significant differences were found between the orders in which communication skills were presented, and compared to the control group, up to 50% of women were capable of reaching coital orgasm after treatment.

Bibliotherapy, in the form of a self-help manual, was also evaluated as a possible form of treatment. A series of articles published results from a clinical trial that involved 23 couples and was based on the Heiman and LoPiccolo manual treatment [6]. The first article evaluated three components of sex therapy: sensate focus I, sensate focus II and ban on intercourse. Subjects were divided into three therapeutic groups: bibliotherapy with minimal therapist contact, classic couples therapy and group therapy [16]. All of the materials provided to the subjects were also based in the Heiman and LoPiccolo manual. The therapy focused on information about sexuality, sensate focus, sexual skills acquisition, and elimination of performance anxiety. Group therapy was divided into male and female groups. The obtained results noted that sensate focus exercises and the ban on intercourse (found to be source of anxiety to the participants) led to an increase in enjoyment of non-coital sexual activities, although this did not translate into better orgasmic responsiveness.

A third study based on the same manual compared therapeutic formats [17]: Standard Couples Therapy (one hour per week with a therapist for 14 weeks); Group Therapy (eight women, and partners only participated at three time points: beginning, middle and end of therapy); and bibliotherapy with minimal therapist contact (two sessions, one at the beginning of treatment and the other at the end). The results showed that all three formats improved sexual function and quality, but the format with the best results was standard couple’s therapy. Women in this format reported great satisfaction with foreplay, higher orgasmic responses and satisfaction with communication and affection. Later on, in a complementary study [18], the authors concluded that affective and cognitive measures such as satisfaction and happiness had improved more than behavioral measures, suggesting that sex therapy can lead to cognitive affective changes even in the absence of behavioral changes.

Still based on the Heiman and LoPiccolo manual, another clinical trial was conducted. Forty-three couples with orgasmic disorder complaints were divided into two groups: one with minimal therapist contact (MCT), with only monthly sessions; and one with full therapist contact (FCT), with weekly sessions. The results suggest that the MCT group had better results. Women in this group learned how to use a vibrator and had better orgasmic responses. The authors believed that, once the subjects had the sense of controlling their treatment, they would engage and present with better outcomes [19].

Thirteen women, diagnosed with orgasmic dysfunction (both primary and secondary) were randomly assigned to minimal contact with therapist or control group, using Heiman and LoPiccolo manual. During 7 weeks, they had 3 ½ hours individual meeting with the therapist, while the control group received texts about human sexuality. In the treatment group, subjects had improvement in all measures. Follow-up showed that the gains were maintained and orgasm frequency were increased [20].

Using a behavioral protocol, researchers compared individual versus group intervention in primary and secondary orgasmic dysfunction. Fifty women were divided in two groups and then assigned to group, individual or waiting-list control (WLC) group. In both treatment conditions (group and individual) women had better outcomes than WLC, but women diagnosed as primary orgasmic dysfunction had worse outcomes, with only 25% reporting achieving orgasm [21].

Pelvic physical exercises and their impact on female sexual function were also assessed. The first clinical trial published compared two groups of women: one instructed on Kegel’s pelvic exercises; the other received information on sexual awareness, respiratory training and muscle relaxation [22]. The objective was to determine if these exercises, designed to increase tonus of pelvic muscles, could help improve orgasmic responsiveness in non-orgasmic women. Twelve women, who could reach orgasm through masturbation but not coitus, participated in the study. Both groups were instructed to perform exercises at home for 20 minutes a day for eight weeks. The results did not support the hypothesis that orgasmic response would improve, although muscular strength was achieved. The women in the relaxation group had higher rates of sexual satisfaction at the end of treatment. Another study found similar results [23], the women showed improvement in muscular strength but not in orgasmic response; however, it was noted that it was difficult for women to comply with the daily twenty minute exercise period.

A study evaluated cognitive-behavioral therapy (CBT) for women with complaints of hypoactive sexual desire disorder (HSDD) [24] Seventy-four couples were separated into two groups: group CBT and waiting list control. The CBT program had 12 weeks with two-hour sessions and each group had 4 to 6 couples and the sessions were conducted by a couple of therapists. Using techniques such as psychosexual education, sensate focus, communication skills, positive reinforcement, cognitive restructuring and sexual fantasies training, women who underwent the program had an improvement of sex life. After therapy, 74% of women were considered cured. In comparison with the control group, couples in the CBT group reported greater satisfaction with marital and sexual life. Only three couples abandoned treatment.

Sexual pain problems have received some attention from researchers. A study with women suffering from vaginismus [25] compared three groups: 3 month CBT program; bibliotherapy; and waiting list control group. One hundred and seventeen women were distributed among the groups. The treatment was considered successful if the women had full penile penetration after therapy. From 83 women in CBT group, 27 (33%) reported full penetration. Those women also reported less fear of coitus at the end of treatment. Another study compared CBT and supportive therapy in the treatment of vulvodynia [26]. Vulvodynia is a gynecological condition characterized by vulvar pain and is often accompanied by sexual pain disorders. Fifty women participated in the study, but only 42 finished the treatment. The CBT protocol was adapted from a chronic pain protocol and involved techniques such as relaxation; supportive therapy was non-directive, involved no behavioral intervention and focused on patients expressing their feelings. The results suggested that both approaches were effective, with 41.7% of women reporting a 33% reduction in pain scores.

CBT was also compared to topical treatment with cream [27]. Ninety-seven women with vestibulodynia, a common type of sexual pain, characterized by burning or cutting sensations in the entrance of the vagina, were randomized into two groups: CBT and topical treatment with a corticosteroid cream. At the end of treatment, both groups showed improvements in pain scores, but at follow-up, the CBT group continued to show improvement. The authors also presented the information that women who had higher scores for pain self-efficacy prior the treatment had better results. The CBT goal of reducing fear-avoidance behaviors and anxiety, promoting a better understanding of sexuality and improving a sense of self-efficacy seem to play an important role in the treatment of vestibulodynia.

A cognitive behavioral bibliotherapy approach was evaluated by a Netherlands group [28]. One hundred and ninety-nine couples with different sexual dysfunction complaints were randomized into two groups: CBT bibliotherapy with minimum therapist contact by telephone and a waiting list control group. Patients received a manual with CBT techniques and were instructed by a researcher in how to use it. They were encouraged to read the chapters they believed had some relation to their complaints. Compared to the control group, the treatment group had greater improvements and women with vaginismus seemed to have the greatest benefits from the program. Both men and women reported a better quality of sex life.

One study evaluated psychological and pharmacological treatments combined [29]. Forty-eight couples were randomized into eight possible combinations from among the following options: testosterone or placebo treatment; sex therapy with monthly or weekly sessions; a female therapist or female–male couple of therapists. The couples received 3 months of treatment. The sex therapy was based on Masters and Johnson and Heiman protocols. Testosterone doses were 10 mg sublingual. The results suggested that the treatment with the best outcomes was placebo plus weekly sessions, followed by testosterone plus monthly sessions.

A group of researchers evaluated the impact of marital therapy prior to sex therapy [30]. There were two groups; one received marital therapy while the other received placebo sex therapy. After nine sessions, classical sex therapy was initiated. The results suggested that both groups benefited, but in the group with marital plus sex therapy, the results were more consistent, and the couples showed higher levels of sexual desire comparable to that of non-dysfunctional couples. Their marital adjustment also improved. The researchers concluded that in marital therapy, focusing on better communication, conflict resolution and assertiveness may enhance the effects of sex therapy.

Recently, a group studied the reliability of an internet-based treatment program for sexual dysfunctions [31]. Thirty-nine women were divided into 2 groups: treatment and control. The treatment group used Revive, a program based on CBT techniques and had unlimited access to a therapist via e-mail, for ten weeks and with five modules. Each module has a list of goals that had to be attained to proceed with treatment. The results showed improvements in the women’s scores of sexual function, but not all reported a complete reversal of symptoms. Thus, Revive can be a suitable tool for treatment, but further analysis is needed.

Recently, many researchers have investigated mindfulness training and its impact on psychiatric disorders, including sexual dysfunctions. Women from a non-clinical population and in meditation program training were compared to a control group and showed faster responses to sexual stimuli, suggesting that these exercises improve the ability of perceive physiological responses. They also showed improvements in attention and self-judgment [32].

## Discussion

Prevalence studies indicate that 40-45% of women experience some form of sexual dysfunction in life [1] and about 25% have some level of distress related to this condition [33]. These rates justify the interest that has been paid to sexual function in the last years. Researchers have been discussing the applicability of the current model for human sexual response for women’s sexuality. Many have theorized new models taking in account aspects known to be important for females [34]. And Basson’s model, which postulates that other factors rather than spontaneous desire play an important role in women’s engagement to sexual activities, seems to be more applicable to women, especially those in a long-term relationship [35]. The new understanding of female sexuality brought up issues related to the diagnosis of their dysfunction. For DSM-V, a review of all disorders was proposed and the inclusion of a new category was made [36-40].

But despite all the discussion about diagnostic criteria, there’s much to improve in sexual dysfunction treatment. Drug therapy has not reached a consensus, although some studies pointed out bupropion as an alternative drug that seems to improve sexual function, when compromised.

When it comes to sex therapy, Masters and Johnson [5] and Heiman and LoPiccolo [6] protocols are still the most used for treating sexual problems, which brings up a question: if there’s an effort to understanding female sexuality, why this effort is not employed to improve sex therapy?

It seems that sex therapy has an efficacy in treating some disorders and has a positive effect in sexual function, improving sex life. But the current literature has not clearly demonstrated that is efficient to all sexual problems seen in clinical settings.

As seen in this review, the focus of sex therapy seems to be on the expected result of sex: orgasm. For a long time, the main goal was help women to improve their capacity to obtain orgasm. Other issues well addressed in literature are sexual pain disorders. Vaginismus and dyspaurenia, two problems that prevent women from engaging in sex. Strategies to help women achieve full penile penetration have been developed and applied. Other problems such as lack or diminished sexual desire, one of the most frequent complaints among women, have little empirical evidence that sex therapy is effective in its treatment.

Another important issue is the lack of studies addressing homosexual individuals. All the studies included in this review targeted heterosexual couples, and some even excluded those with homosexual orientation. In order to establish sex therapy efficacy, why not include them in the trials?

Since anxiety was given an important role in etiology of sexual dysfunction, CBT has been used in their treatment. CBT is known to provide changes in thinking and beliefs that might affect how the individual behaves towards sex. Changing these beliefs would reduce anxiety related to sex and performance. Adding to these changes, behavioral strategies could improve quality of sex life. And when it comes to sexual dysfunctions, CBT has positive outcomes. One study even pointed out that CBT for other issues may be beneficial to sexual problems [41].

The heterogeneity of sexual dysfunctions can difficult the establishment of a treatment protocol, demanding more studies addressing specific dysfunctions, in order to evaluate which techniques are more effective to each problem. Orgasmic and sexual pain disorders are the most extensively studied disorders and those in which sex therapy seems to have better outcomes.

This paper points out the need to systematically evaluate sex therapy for all sorts of sexual disorders, populations, searching for the most effective interventions and establishing the treatment efficacy, when compared to other forms of treatment.

## Competing interests

The authors declared that there are no competing interests.

## Authors’ contributions

VMP, OAC, AEN and ACS participated in the definition of the study design and the protocol. Authors VMP, OAC, SM, AEN and ACS managed the literature searches. Authors VMP, OAC, SM, AEN and ACS wrote the first draft of the manuscript. All authors contributed to and have approved the final manuscript.
